# Gain-of-Function STIM1 L96V Mutation Causes Myogenesis Alteration in Muscle Cells From a Patient Affected by Tubular Aggregate Myopathy

**DOI:** 10.3389/fcell.2021.635063

**Published:** 2021-02-26

**Authors:** Elena Conte, Alessandra Pannunzio, Paola Imbrici, Giulia Maria Camerino, Lorenzo Maggi, Marina Mora, Sara Gibertini, Ornella Cappellari, Annamaria De Luca, Mauro Coluccia, Antonella Liantonio

**Affiliations:** ^1^Department of Pharmacy—Drug Sciences, University of Bari, Bari, Italy; ^2^Neuromuscular Diseases and Neuroimmunology Unit, Foundation IRCCS Neurological Institute C. Besta, Milan, Italy

**Keywords:** STIM1, tubular aggregate myopathy, myogenesis (*in vitro*), high content imaging, calcium homeostasis

## Abstract

Tubular Aggregate Myopathy (TAM) is a hereditary ultra-rare muscle disorder characterized by muscle weakness and cramps or myasthenic features. Biopsies from TAM patients show the presence of tubular aggregates originated from sarcoplasmic reticulum due to altered Ca^2+^ homeostasis. TAM is caused by gain-of-function mutations in STIM1 or ORAI1, proteins responsible for Store-Operated-Calcium-Entry (SOCE), a pivotal mechanism in Ca^2+^ signaling. So far there is no cure for TAM and the mechanisms through which *STIM1* or *ORAI1* gene mutation lead to muscle dysfunction remain to be clarified. It has been established that post-natal myogenesis critically relies on Ca^2+^ influx through SOCE. To explore how Ca^2+^ homeostasis dysregulation associated with TAM impacts on muscle differentiation cascade, we here performed a functional characterization of myoblasts and myotubes deriving from patients carrying STIM1 L96V mutation by using fura-2 cytofluorimetry, high content imaging and real-time PCR. We demonstrated a higher resting Ca^2+^ concentration and an increased SOCE in STIM1 mutant compared with control, together with a compensatory down-regulation of genes involved in Ca^2+^ handling (*RyR1, Atp2a1, Trpc1)*. Differentiating STIM1 L96V myoblasts persisted in a mononuclear state and the fewer multinucleated myotubes had distinct morphology and geometry of mitochondrial network compared to controls, indicating a defect in the late differentiation phase. The alteration in myogenic pathway was confirmed by gene expression analysis regarding early (*Myf5, Mef2D*) and late (*DMD, Tnnt3*) differentiation markers together with mitochondrial markers (*IDH3A, OGDH)*. We provided evidences of mechanisms responsible for a defective myogenesis associated to TAM mutant and validated a reliable cellular model usefull for TAM preclinical studies.

## Introduction

In its primary form, Tubular Aggregate Myopathy (TAM) is a clinically heterogeneous and very rare skeletal muscle disorder, in most cases inherited in an autosomal dominant pattern (Böhm and Laporte, 2018; Michelucci et al., 2018; Morin et al., 2020). Signs and symptoms typically begin in childhood and worsen over time. TAM patients can be characterized by asymptomatic elevated creatine kinase (CK) levels as well as by muscle weakness predominantly affecting the proximal muscles of lower limbs. Myalgia and cramps have also been described (Böhm et al., 2014; Hedberg et al., 2014; Walter et al., 2015) and in some cases the full picture of the multisystemic Stormorken syndrome develops (Morin et al., 2020). A consistent histopathological feature of TAM patients is represented by the presence of tubular aggregates (TAs) in skeletal muscle fibers. TAs are formed by regular arrays of densely packed membrane tubules, most likely originating from sarcoplasmic reticulum (SR) (Böhm et al., 2013, 2017; Endo et al., 2015; Harris et al., 2017; Böhm and Laporte, 2018). However, TAs represent a non-specific morphological alteration being present in several neuromuscular disorders associated to SR stress (Michelucci et al., 2018).

TAM can be caused by heterozygous mutations in STIM1 or ORAI1 gene (Morin et al., 2020), both encoding for Ca^2+^ homeostasis key regulators, and *CASQ1* gene (Barone et al., 2017; Böhm et al., 2018), encoding for calsequestrin, the major Ca^2+^ buffering protein in skeletal muscle SR. Particularly, STIM1 and ORA1 are key components of the calcium release-activated calcium (CRAC) channels (Prakriya, 2009), which are activated following intracellular SR or endoplasmic reticulum (ER) Ca^2+^ store depletion and allow extracellular Ca^2+^ influx through a process called store operated Ca^2+^ entry (SOCE). SOCE is therefore fundamental in a variety of cellular functions, including secretion, transcription, motility, enzyme activity, Ca^2+^ store filling-state, and muscle contraction (Kiviluoto et al., 2011; Stiber and Rosenberg, 2011; Cho et al., 2017). SOCE-dependent Ca^2+^ signaling is also crucial for the onset of skeletal muscle development (Stiber et al., 2008; Darbellay et al., 2009, 2010; Li et al., 2012; Phuong et al., 2013; Wei-Lapierre et al., 2013; Tu et al., 2016). SOCE is indeed necessary for the activity of various Ca^2+^-dependent enzymes regulating myogenesis-associated transcription factors, as has been shown for Nuclear Factor of Activated T cells (NFAT) in murine models (Kegley et al., 2001; Armand et al., 2008), as well as for Myocyte Enhancer Factor-2 (MEF2) and Myogenin in myoblasts deriving from human biopsies (Louis et al., 2008; Darbellay et al., 2009, 2010).

STIM1 is an ER/SR transmembrane protein activated by a drop in ER/SR calcium levels. This is at the basis of the SOCE process. Indeed, following Ca^2+^ store depletion, the STIM1 Ca^2+^-sensing intraluminal EF-hands undergo a conformational switch leading to protein di- and oligomerization, to an extended active state, interacting and activating the plasma membrane Ca^2+^ channel ORAI1, thus finally leading to Ca^2+^ entry (Park et al., 2009; Cho et al., 2017; Gudlur et al., 2018).

To date, 18 distinct STIM1 mutations causing TAM have been identified, of which 15 cluster in the EF-hand domains (Morin et al., 2020). According to the resolved STIM1 protein structure (Yang et al., 2012; Zhu et al., 2017; Lopez et al., 2020), TAM-associated STIM1 EF-hand mutations appear to affect amino acids involved in Ca^2+^ coordination or forming a hydrophobic pocket which maintains STIM1 in a folded state (Stathopulos et al., 2008; Böhm et al., 2014). Functional impact of TAM-associated STIM1 EF-hand mutations has been investigated through their heterologous expression in murine C2C12 myoblasts (Böhm et al., 2013) as well as in other engineered cell lines (Hedberg et al., 2014; Nesin et al., 2014). All known EF-hand mutations were shown to induce STIM1 oligomerization and clustering independently from intraluminal SR/ER Ca^2+^ level, thus indicating a constitutive STIM1 and SOCE activation and a gain-of-function effect leading to intracellular Ca^2+^ accumulation (Böhm and Laporte, 2018). Importantly, only in selected cases, *i.e*., the STIM1 A84G (Böhm et al., 2013) and G81A (Walter et al., 2015) mutations, Ca^2+^ homeostasis alteration was directly confirmed on TAM patient-derived muscle cells.

So far, there is no cure for TAM and there is little information in literature regarding a therapy or management of this disorder. Considering that symptoms generally occur at a young age and significantly reduce the quality of life of the affected people, there is an urgent need to find new treatments. The resulting aberrant Ca^2+^ homeostasis associated to TAM mutants is likely the key cellular event causing serious damage on muscle development and integrity. Thus, the investigation of cellular processes dysfunction induced by Ca^2+^ homeostasis alteration associated with TAM mutants remains a pivotal strategy to identify novel druggable targets for this rare disease.

In this study, the STIM1 L96V-associated Ca^2+^ homeostasis dysregulation has been evaluated in patient-derived skeletal muscle cells. This mutation, located in the Ca^2+^ sensing canonical EF hand (cEF-hand) of STIM1 protein, was identified in a 13 year old girl showing CK elevation and lower limb weakness and myalgia, along with a muscle histology characterized by TAs, fiber size variability and internal nuclei (Böhm et al., 2014). Functional and morphological characterization of patient derived STIM1 L96V myoblasts and myotubes has been performed by using Ca^2+^ cytofluorimetry, molecular biology and high content imaging technologies. Our results confirm the STIM1 L96V-associated Ca^2+^ homeostasis dysfunction on patient-derived cells and demonstrate, for the first time, that the STIM1 L96V mutation alters the myogenic differentiation program, particularly regarding the terminal differentiation step, thereby providing new insights in the pathogenesis of STIM1-related TAM.

## Materials and Methods

### Cell Culture

Human muscle samples were provided by the Telethon biobank at Besta Neurological Institute in Milan. Written, informed consent was obtained from the subjects or their parents/legal guardians. Research was conducted according to protocols approved by the Institutional Review Board of the Besta Neurological Institute and University of Bari, and in compliance with the Helsinki Declaration and local legislation. Particularly, we used myoblasts and myotubes deriving from one TAM patient's biopsy carrying STIM1 L96V mutant. The patient's mutation was found by Böhm's laboratory in 2014 (Böhm et al., 2014). Particularly, patient' cells genetic characterization was performed on DNA extracted from blood cells. Sanger-sequencing has been used for all coding exons and the adjacent splice-relevant regions of STIM1 (NM_001277961). The mutation found in the proband was also analyzed in the parents (our patient belongs to Family 3 in Böhm et al., 2014). The patient carries a *de novo* heterozygous mutation. Mutations in DMD and lamin A/C (LMNA) were also exclude for the patient. A control muscle cell line was obtained from a patient not affected from TAM (who had no STIM, ORAI, or CASQ1 mutations) of the same age and sex of affected patients. To assess the myogenesis characteristics in control condition, gene expression analysis of genes involved in the differentiation of myoblasts to myotubes was performed both in cells deriving from a patient not affected from TAM (control) and for comparison in a control muscle cell line (HMb_2) (see Supplementary Material). Myoblast derived cells were isolated from patients' biopsy, cultured using the protocol described in Zanotti et al. (2007) and used under 2 different stages of differentiation. Primary myoblasts were derived directly from biopsied material by culturing in Dulbecco's modified Eagle's medium (DMEM; Lonza Group Ltd, Basel, Switzerland) containing 20% heat-inactivated fetal bovine serum (FBS) (Gibco Life Technologies), 1% penicillin-streptomycin (Lonza), L-glutamine (Lonza), 10 μg/ml insulin (Sigma Aldrich, St. Louis, MO), 2.5 ng/ml basic fibroblast growth factor (bFGF) (Gibco Life Technologies), and 10 ng/ml epidermal growth factor (EGF) (Gibco). Cells were grown on plastic. The medium was changed twice weekly and the cultures examined by inverted-phase microscopy. Once at 70% confluence, they were dissociated enzymatically with trypsin-EDTA (Sigma) and seeded for immediate propagation, or frozen in medium containing 10% DMSO (Sigma) for later propagation or other use. To obtain myotubes, the myoblasts were seeded into 35 mm dishes or in chamber slides in DMEM proliferating medium. At 70% confluence, proliferating medium was changed to differentiating medium (DMEM, 1% penicillin-streptomycin, L-glutamine and insulin, without FBS, or growth factors) and the myoblasts could differentiate into myotubes after 10 days (Zanotti et al., 2007). After the isolation, we analyzed phenotypic characteristic of the cell model carrying STIM1 L96V mutant, alongside with the non-mutated counterpart which represents control muscle cell. We evaluated various parameters on the proliferating and the differentiating myoblasts (T1, 5 days) and on the differentiated myotubes (T2, 10 days).

For fluorescence imaging analysis, both control and Leu96Val STIM1 myoblasts were seeded in quadruplicate in 96-well culture plates (96-well CallCarrier^TM^, PerkinElmer) at 6,000/100 μL density and incubated at 37°C and 5% CO_2_ to allow cell growth and proliferation. After 48 h incubation and cell washing, the growth medium was replaced by differentiation medium (T0) and incubation continued for further five (T1) or ten (T2) days, taking care to change the medium every 2 days.

### Cytosolic Calcium Measurements

Myoblasts or myotubes grown on coverslip were loaded for 30 min at room temperature with the cell permeant fluorescent Ca^2+^ indicator 5 μM Fura-2-AM (Molecular Probes-Invitrogen, Italy) mixed to 0.05% (v/v) Pluronic F-127 (Molecular Probes) in normal physiological solution. Ratiometric images of Fura-2 fluorescence were monitored using an inverted Eclipse TE300 microscope (Nikon, Japan) with a 40X Plan-Fluor objective (Nikon, Japan). Fluorescence measurements were made using a QuantiCell 900 integrated imaging system (Visitech International Ltd., Sunderland, UK) as previously described (Conte et al., 2017). During the experiments, pairs of background subtracted images of Fura-2 fluorescence (510 nm) emitted after excitation at 340 and 380 nm were acquired and ratiometric images (340/380 nm) were calculated for each cell using QC2000 software. Subsequently fluorescence ratio values were converted to the resting cytosolic calcium concentration, [Ca^2+^]_i_ (nM), after a calibration procedure using the following equation: [Ca^2+^]_i_ = (R-R_min_)/(R_max_-R)^*^${\text{K}}_{\text{D}}^{*}$β where R is the ratio of the fluorescence emitted after excitation at 340 nm to the fluorescence after excitation at 380 nm; K_D_ is the affinity constant of fura-2 for calcium, which was taken as 145 nM (Molecular Probes); and β is a parameter according to Grynkiewicz et al. (1985) that was determined experimentally *in situ* in ionomycin-permeabilized muscle fibers as previously described (Conte et al., 2017).

To measure SOCE, 2 μM Thapsigargin was used to passively deplete the Ca^2+^ stores in the calcium free-solution and then extracellular Ca^2+^ was applied to myoblasts or myotubes (SOCE protocol). The normal physiological solution was composed of 148 mM NaCl, 4.5 mM KCl, 2.5 mM CaCl_2_, 1 mM MgCl_2_, 0.44 mM NaH_2_PO_4_, 12 mM NaHCO_3_, and 5.5 mM glucose. The pH of all solutions was adjusted to 7.3–7.4 by bubbling them with 95% O_2_/5% CO_2_. The calcium free-solution has the same composition of normal physiological solution except that CaCl_2_ was omitted and 10 mM ethylene glycol bis(β-aminoethyl ether)-*N,N,N',N'*-tetracetic acid (EGTA) was added. All chemicals cited above as well as ionomycin, caffeine and thapsigargin were purchased from Sigma (St. Louis, MO, USA).

### Fluorescent Probes, Image Acquisition, and Analysis

Cells were stained with a fluorophore dye cocktail containing Hoechst 33,342 (Thermofisher) for nuclear staining and MitoTracker^®^ Deep Red (Thermofisher), 1 μM and 50 nM final concentration, respectively. MitoTracker^®^ Deep Red enables the detection of cell shape and is also used to evaluate cytoplasmic morphological parameters along with mitochondrial mass and network features (texture). After 30 min incubation with the dye cocktail (37°C, 5% CO_2_), live cell image acquisitions were performed from 16 distinct areas/well using a Perkin Elmer Operetta High Content Imaging system (40x WD objective). Cytoplasmic and nuclear morphological parameters of differentiating control and STIM1 L96V myoblasts at T0, T1, and T2 time points were analyzed at single-cell level using Harmony 3.1 software. Briefly, images were first segmented into nuclei and cytoplasm using “Find Nuclei” and “Find Cytoplasm” Building blocks on Hoechst and MitoTracker^®^ Deep Red channels, respectively. After image segmentation, a set of basic intensity and morphological properties (*e.g*., area, roundness, etc.), and mitochondrial texture patterns of selected objects was calculated using “Calculate Intensity Properties,” “Calculate Morphology Properties” and “SER texture analysis” building blocks, respectively. Distinct mononuclear and multinuclear morphological phenotypes of skeletal muscle cells were manually identified and then used in the PhenoLOGIC^TM^ machine learning module of the Harmony software. The PhenoLOGIC module requires users to supervise training selecting about 100 representative cells/class, thus allowing the software to distinguish different phenotypes. The software performs a linear discriminant analysis to create a linear combination of the most relevant parameters that are then applied to untrained sample wells to classify cells. This approach is very helpful, considering the morphological variety of differentiating skeletal muscle cells. As far as the mitochondrial texture properties are concerned, the analysis was performed using the SER features method. Briefly, the image texture features usually described as smooth, rough, granular, homogeneous/inhomogeneous, linear *etc*. were quantified calculating the numerical properties which quantitatively describe the texture. The SER (Spot, Edge, Ridge) features method includes a set of eight properties (spot, hole, edge, ridge, valley, saddle, bright, and dark) sensitive to distinct intensity patterns according to the property geometry designation. All results are reported as mean ± SD from three independent experiments, each performed in quadruplicate. GraphPad Prism (GraphPad Software, Inc.) was used for calculating statistics and creation of graphs.

### Real Time PCR

Total RNA was extracted from myoblasts and myotubes using RNeasy Micro Kit (Qiagen C.N. 74004, Valencia) according to the manufacturer's protocols and RNA quantity was assessed using a spectrophotometer (ND-1000 NanoDrop, Thermo Scientific, United States). Reverse transcription and real-time PCR analysis were performed as previously described (Conte et al., 2017). The mRNA expression of genes was normalized to the best housekeeping gene: Beta-actin (*Actb)* selected from beta-2-microglobulin (*B2m*) and *ACTB* by Normfinder software. Genes were analyzed by the use of TaqMan Hydrolysis primer and probe gene expression assays that are produced by Life-Technologies with the following assay IDs: *ORAI1* assay ID: Hs03046013_m1; *STIM1* assay ID: Hs00963373_m1; *RYR1* assay ID: Hs00166991_m1; *ATP2A1* (encoding SERCA1 protein) assay ID: HS01092295_m1; *CACNA1S* (encoding Cav1.1 protein) assay ID: Hs00163885_m1; *ATP1A2* (encoding SERCA2 protein) assay ID: Hs00265131_m1; *TRPC1* assay ID: Hs00608195_m1; *TRPC4* assay ID: Hs01077392_m1; *OGDH* assay ID: Hs01081865_m1; *IDH3A* assay ID: Hs01051668_m1; *PAX7* assay ID: Hs00242962_m1; *MYF5* assay ID: Hs00271574_m1, *MYOD1* assay ID: Hs00159528_m1; *MEF2D* assay ID: Hs00954735_m1; *MYOG* assay ID: Hs01072232_m1; *TNNT3* (encoding Troponin protein) assay ID: Hs00952980_m1; *DMD* (encoding Dystrophin protein) assay ID: Hs00758098_m1; *B2M* assay ID: Hs00984230_m1 and *Actb* assay ID: Hs99999903_m1. For genes that were poorly expressed, such as *Trpc1, Trpc4, Myf5*, and *Dmd*, preamplification by TaqMan PreAmp Master Mix (Life Technologies C.N. 4391128) was made before real-time experiments with a set-up of pre-amplification detailed in Conte et al. (2017). The real-time PCR protocols were performed in line with the guidelines for qPCR (Bustin et al., 2009).

## Statistics

Statistical analysis was performed using Student's *t-*test, with *p* < 0.05 or less considered as significant for calcium measurement and gene expression analysis. GraphPad Prism (GraphPad Software, Inc.) was used for calculating statistics (*t*-test, *chi*-square test, analysis of variance and Tukey HSD *post-hoc* test) and creation of graphs for high content fluorescence analysis.

## Results

### Calcium Homeostasis

**Figure 1:**
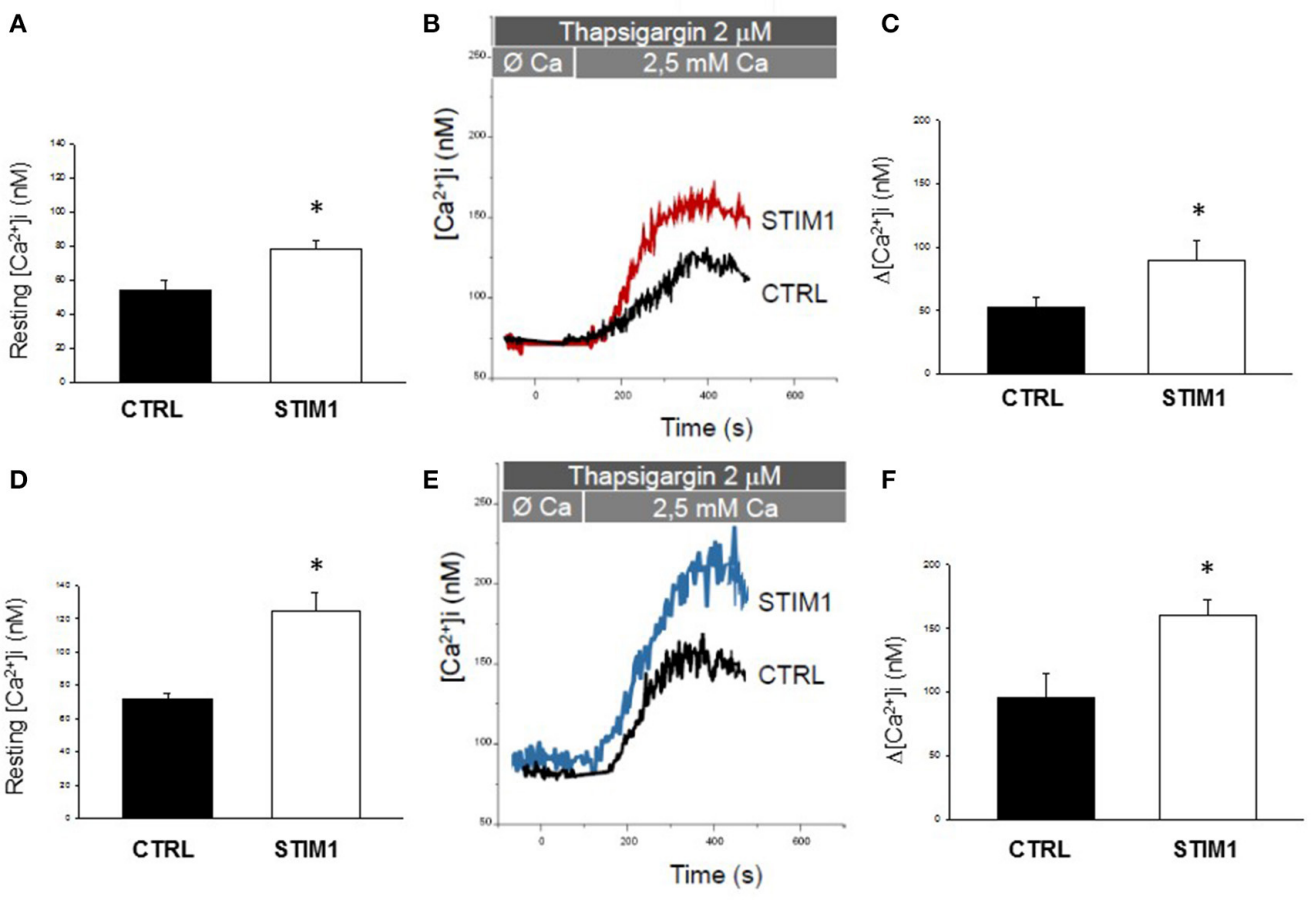
Calcium homeostasis characterization of myoblast and myotubes carrying STIM1 L96V mutation. **(A–D)** Resting intracellular calcium, resting [Ca^2+^]i measured in control and STIM1 L96V myoblasts and control and STIM1 L96V myotubes. Each bar represnts the mean ± SEM of resting [Ca^2+^]i measured in 35–40 cells; **(B–E)** Representative traces of increased Ca^2+^ entry in store depleted thapsigargin treated cells after addition of extracellular calcium (see SOCE protocol described in Material and Methods) in control and STIM1 L96V myoblasts and in control and STIM1 L96V multinucleated myotubes **(C–F)** Amplitude values of [Ca^2+^]i increase observed with SOCE protocol in control and STIM1 L96V myoblasts and in control and STIM1 L96V multinucleated myotubes. Each bar represents the mean ± SEM of [Ca^2+^]i increase measured in 25–30 cells. Statistical significance was determined by unpaired Student's *t-*test, with a value of *P* < 0.05 considered significant, ^*^Significantly different.

In engineered murine myoblasts, all TAM-associated STIM1 EF-hand mutations so far identified, including the L96V one, have been shown to induce protein clustering and excessive Ca^2+^ entry independently from SR intraluminal Ca^2+^ level (Böhm and Laporte, 2018). Particularly, C2C12 myoblasts transfected with STIM1 L96V-YFP displayed statistically significant STIM1 clustering regardless of SR Ca^2+^ depletion (Böhm et al., 2014). Here, Ca^2+^ homeostasis alterations associated with STIM L96V mutation have been investigated on skeletal muscle cells deriving from TAM patient's biopsy. The clinical, histological and functional phenotype of this patient has been already defined (Böhm et al., 2014). Free intracellular Ca^2+^ was measured by using the ratiometric fluorescent dye Fura-2. Both STIM1 L96V myoblasts and myotubes, the latter obtained after 10 days in differentiation medium, showed higher basal cytoplasmic Ca^2+^ level with respect with control cells (Figures 1A,D).

To examine the effect of STIM L96V mutation on SOCE, Ca^2+^ was first depleted from the SR of myoblasts or myotubes with thapsigargin, in the absence of extracellular Ca^2+^, to avoid extracellular Ca^2+^ entry during depletion, and then extracellular Ca^2+^ was applied to myoblasts or myotubes to measure SOCE (Figures 1B,E, respectively). Both myoblasts and myotubes carrying STIM1 L96V mutation displayed a significant augmented SOCE compared to the respective control cells (Figures 1B,C,E,F).

Furthermore, to assess the Ca^2+^ amount in the SR, we treated STIM1 L96V myotubes with 40 mM caffeine to induce extensive store depletion. Importantly, more Ca^2+^ was released from the SR in STIM1 L96V myotubes than in control myotubes [Caffeine-induced Δ[Ca^2+^]i = 320 ± 27 nM and 214 ± 35 nM in mutant and control, respectively], thus demonstrating also an increase in the Ca^2+^ SR content of STIM1 L96V muscle cells.

Thus, our functional assay conducted for the first time on patient-derived myoblasts and myotubes revealed that STIM1 L96V mutation is consistent with SOCE constitutive activation, i.e., with a gain of function effect.

### High Content Imaging of Differentiating Skeletal Muscle Cells

Different lines of evidence indicate that SOCE is a key factor controlling myoblasts fate (Louis et al., 2008; Darbellay et al., 2009, 2010; Michelucci et al., 2018) as well that mitochondria participate in the differentiating process of various cell types, including muscle cells (Noguchi and Kasahara, 2018). On this basis, cellular morphology properties and mitochondrial network features of control and STIM1 L96V differentiating cells have been investigated *in vitro* by automated fluorescence microscopy. The quantitative analysis of morphological changes associated with differentiation was performed on living myoblasts, at distinct time points after substitution of differentiation medium for growth medium (T0, T1, and T2, corresponding to 0, 5, and 10 days, respectively).

#### Differentiation-Associated Morphological Features

**Figure 2:**
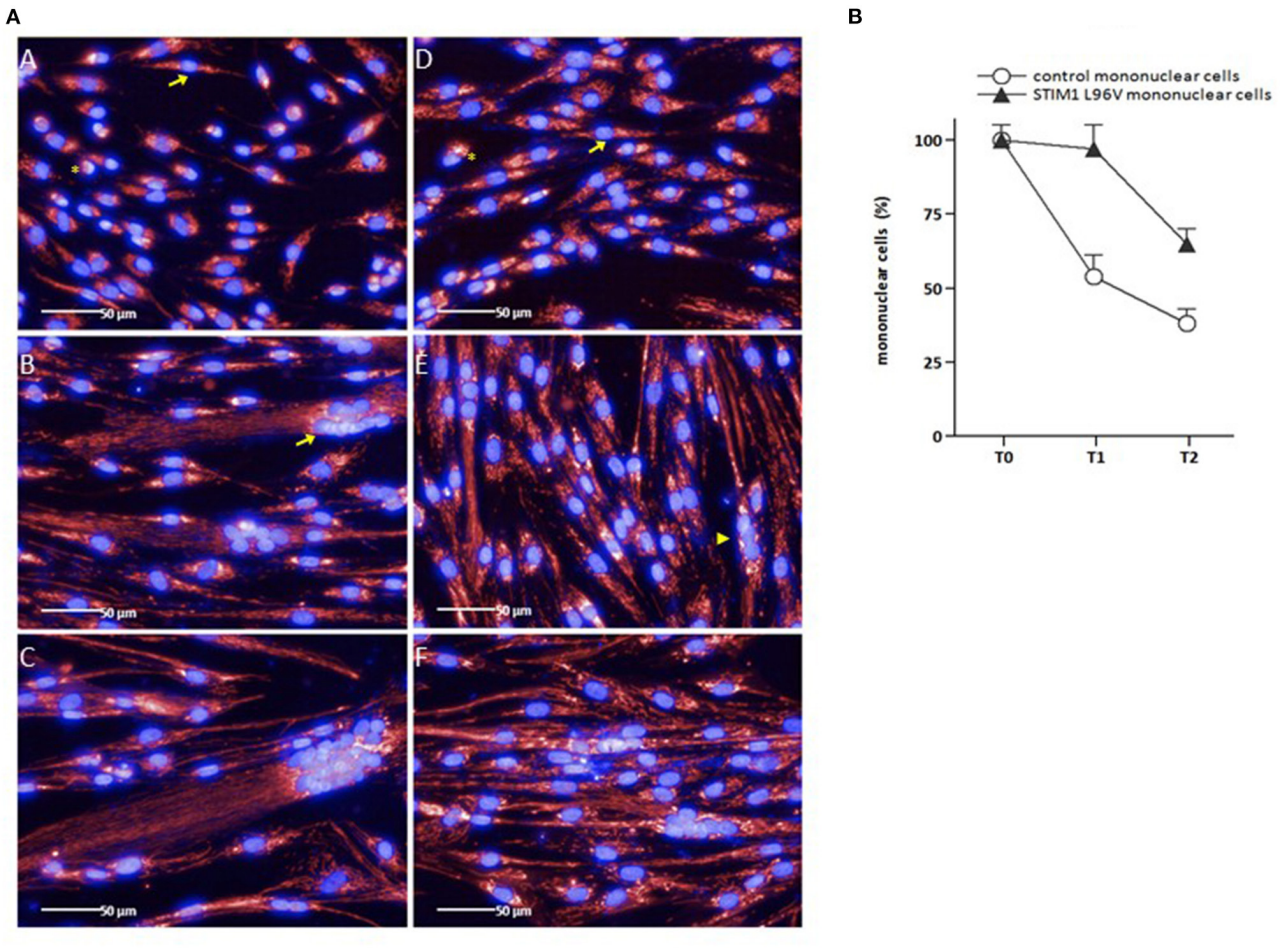
Representative images of control and STIM1 L96V skeletal muscle cells at different time points in differentiation medium **(A)**, and differentiation-associated decrease of control and STIM1 L96V mononuclear cells **(B)**. **(A)** Control and STIM1 L96V mononuclear mononuclear cells were seeded in 96-well cell culture plates (6,000/100 μL). Upon reaching sub-confluence (~60%), growth medium was replaced by differentiation medium to induce myoblast differentiation and myotube formation. For fluorescence staining of mitochondria (red) and nuclei (blue), MitoTracker Deep Red and Hoechst 33,342 were added into the media (final concentration 50 nM and 1 μM, respectively). After 30 min of incubation at 37°C, 5% CO_2_, live-cell images were recorded by automated fluorescence microscopy at day 0 (T0), 5 (T1), and 10 (T2) for control (panels A, B, C) and STIM1 L96V cells (panels D, E, F), respectively (Perkin Elmer Operetta High-Content Imaging System, 40x LWD objective). Asterisks and arrows in panels A and D indicate rounded and spindle-shaped control and STIM1 L96V myoblasts at T0, respectively. The arrow and arrowhead in panels B and E indicate a multinucleated control and STIM1 L96V cell at T1, respectively. **(B)** The percentage of mononuclear control and STIM1 L96V cells at distinct time points in differentiation medium is shown as mean ± SD.

As shown in Figure 2A (panels A–C), the myogenic differentiation process of control myoblasts is characterized by the presence of mononuclear cells at T0, followed by the increase of cell size as well as the emergence of multinuclear cells after 5 days (T1) and, to a greater extent, after 10 days (T2) in differentiation medium. In parallel with the appearance of multinucleated elements, the number of control mononuclear cells progressively decreases (Figure 2B). In the case of STIM1 L96V cells, only rare multinucleated elements are present at T1, containing two-three nuclei [Figure 2A (panel E), arrowhead], whereas elements with more than 10 nuclei are already formed by control cells at this time [Figure 2A (panel B), arrow]. In parallel, mononuclear STIM1 L96V cells show a longer persistence, only at T2 their number being significantly reduced (Figure 2B).

#### Cell Morphometry and Mitochondrial Texture Properties of Mononuclear Control and STIM1 L96V Cells (T0)

Both control and STIM1 L96V myoblasts at T0 are mononuclear cells, characterized by variable size and rounded or spindle-shaped morphology (Figure 2, asterisks and arrows, respectively, in panels A and D). The relative amount of rounded and spindle-shaped myoblasts in the two cell populations was calculated by using the PhenoLOGIC module, finding that the percentage of spindle-shaped cells in STIM1 L96V myoblasts was significantly higher than that of normal cells (47 and 38%, respectively; *p* = 0.005, chi-square test; data not shown). The comparative analysis of cell morphometry parameters reveals the features of control and STIM1 L96V myoblasts at T0 (Table 1). STIM1 L96V myoblasts are about 20% bigger that normal myoblasts, as indicated by cell area values, and have a more pronounced spindle-shaped morphology (roundness index: 0.68 and 0.61, respectively). The mitochondrial mass mean concentration (mitotracker intensity mean) of control and STIM1 L96V myoblasts is similar, whereas the total mitochondrial mass (mitotracker intensity sum) of STIM1 L96V myoblasts is higher, according to their increased cell size.

**Table 1:** Morphological features of control and STIM1 L96V myoblasts at T0.

**Cell morphometry**	**Control myoblasts**	**STIM1 L96V myoblasts**	***P-*value (unpaired *t-*test)**
Cell area (μm^2^)	409 ± 227	506 ± 274	<0.0001
Cell roundness^*^	0.68 ± 0.14	0.61 ± 0.15	<0.0001
Mito intensity Mean (AU)	256 ± 122	261 ± 98	0.09
Mito intensity Sum (AU)	1,017,364 ± 750,531	1,341,283 ± 894,914	<0.0001
Nuclear area (μm^2^)	120 ± 41	141 ± 46	<0.0001
Nuclear roundness^*^	0.944 ± 0.038	0.946 ± 0.038	0.007
Nuclear Intensity Mean (AU)	1,118 ± 261	1,117 ± 236	0.87
Nuclear Intensity Sum (AU)	2,071,623 ± 596,194	2,440,722 ± 641,465	<0.0001

* *Cell and nuclear roundness are proportional to the square root of the area divided by the circumference: it is normalized to give 1 for a perfect circle and decreases for elongated objects. AU, Arbitrary Units. All data are expressed as mean ± SD of measurements performed at single-cell level (n > 5,000 cells/group)*.

**Figure 3:**
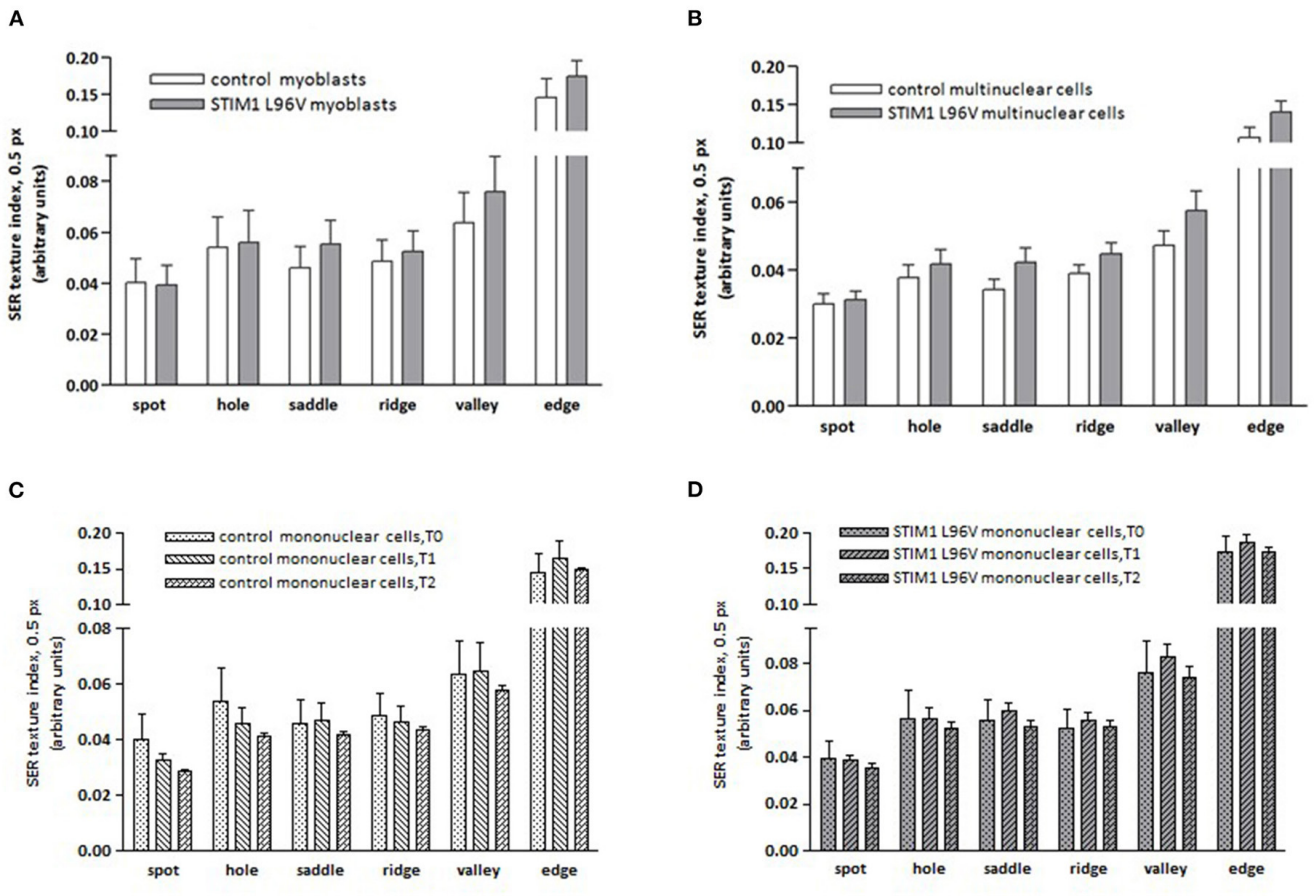
Mitochondrial SER texture indexes of control and STIM1 L96V skeletal muscle cells. **(A)** SER texture indexes of SER spot, hole, saddle, ridge, valley, and edge of normal and STIM1 L96V mononuclear cells at T0 are shown as mean ± SD (cell number > 5,000/group). All SER features of STIM1 L96V myoblasts are significantly different from those of control myoblasts (*p* < 0.0001, unpaired *t-*test), except for spot and hole. **(B)** SER texture indexes of SER spot, hole, saddle, ridge, valley, and edge of normal and STIM1 L96V multinuclear cells are shown as mean ± SD (cell number > 300). All variations of SER texture indexes are significantly different (*p* < 0.005 for spot; *P* < 0.0001 for the remaining filters, unpaired *t-*test). **(C,D)** Differentiation-associated variations of mitochondrial SER texture indexes of control **(C)** and STIM1 L96V **(D)** mononuclear cells; the SER texture indexes at distinct time points in differentiation medium are shown as mean ± SD (cell number>5,000 for T0 groups, and >3,000 for T1 and T2 groups). Time point-associated variations of SER texture indexes are significantly different for all SER features: *P* < 0.0001, ANOVA and Tukey HSD *post-hoc* test, in both control **(C)** and STIM1 L96V **(D)** mononuclear cells, except for hole in the T0–T1 comparison of STIM1 L96V cells.

The effects of STIM1 L96V mutation on the mitochondrial architecture have been evaluated calculating the frequency of texture feature or combination of features using the “SER features” building block of the Harmony software (Supplementary Material). All SER texture indexes of STIM1 L96V myoblasts were significantly different from those of control myoblasts, except for spot and hole feature (Figure 3A). The major differences concern in the order saddle, valley, edge and ridge, indicating that STIM1 L96V myoblasts at T0 are characterized by a more elongated and networked mitochondrial architecture with respect to control myoblasts.

#### Differentiation-Associated Modifications of Mononuclear Control and STIM1 L96V Cells

The differentiation-associated modifications of mononuclear cell morphometry of control and STIM1 L96V cells after 5 (T1) and 10 (T2) days in differentiation medium are reported in Tables 2, 3, respectively. The cell size of control cells progressively increases as well as the spindle-shaped morphology, the major increment occurring in the T0–T1 interval. Moreover, both mitotracker mean intensity and sum intensity values progressively increase, indicating a corresponding enhancement of mitochondrial mass and concentration (Table 2). As far as morphological modifications accompanying STIM1 L96V mononuclear cell differentiation are concerned, the results resemble those of control cells regarding size, spindle-shaped morphology and mitochondrial mass increase (Table 3). In contrast to control cells, however, the mitotracker mean intensity remains substantially unmodified from T0 to T2, suggesting that mitochondrial activity of mononuclear STIM1 L96V cells does not increase along with differentiation.

**Table 2:** Differentiation-associated modifications of control mononuclear cells.

**Cell morphometry**	**T0**	**T1**	**T2**	***P-*value (ANOVA, and Tukey HSD *post-hoc* test)**
Cell area (μm^2^)	409 ± 223	776 ± 56	944 ± 80	<0.0001 (T2>T1>T0)
Cell roundness^*^	0.68 ± 0.14	0.48 ± 0.039	0.44 ± 0.0168	<0.0001 (T0>T1>T2)
Mito intensity Mean (AU)	256 ± 122	359 ± 54	421 ± 20	<0.0001 (T2>T1>T0)
Mito intensity Sum (AU)	1,017,364 ± 750,531	3,214,531 ± 783,315	5,103,299 ± 530,463	<0.0001 (T2>T1>T0)
Nuclear area (μm^2^)	120 ± 41	151 ± 4	148 ± 6	<0.0001 (T1, T2>T0)
Nuclear roundness^*^	0.944 ± 0.038	0.946 ± 0.04	0.945 ± 0.05	0.2
Nuclear Intensity Mean (AU)	1,118 ± 261	2,013 ± 50	1,997 ± 56	<0.0001 (T1, T2>T0)
Nuclear Intensity Sum (AU)	2,071,623 ± 596,194	4,803,092 ± 85,521	4,764,357 ± 88,105	<0.0001 (T1, T2>T0)

* *Cell and nuclear roundness are proportional to the square root of the area divided by the circumference: it is normalized to give 1 for a perfect circle and decreases for elongated objects. AU, Arbitrary Units. All data are expressed as mean ± SD of measurements performed at single-cell level (n > 5,000 cells for T0, and > 3,000 cells/group for T1 and T2 groups)*.

**Table 3:** Differentiation-associated modifications of STIM1 L96V mononuclear cells.

**Cell morphometry**	**T0**	**T1**	**T2**	***P-*value (ANOVA, and Tukey HSD *post-hoc* test)**
Cell area (μm^2^)	506 ± 274	749 ± 44	943 ± 61	<0.0001 (T2>T1>T0)
Cell roundness^*^	0.61 ± 0.15	0.4548 ± 0.0197	0.3942 ± 0.0181	<0.0001 (T0>T1>T2)
Mito intensity Mean (AU)	263 ± 98	265 ± 64	268 ± 58	0.09
Mito intensity Sum (AU)	1,341,283 ± 894,914	2,147,071 ± 365,087	3,292,538 ± 519,703	<0.0001 (T2>T1>T0)
Nuclear area (μm^2^)	141 ± 46	168 ± 23	172 ± 36	<0.0001 (T1, T2>T0)
Nuclear roundness^*^	0.946 ± 0.038	0.950 ± 0.002	0.9339 ±0.0058	0.2
Nuclear Intensity Mean (AU)	1,117 ± 236	1,818 ± 126	1,734 ± 135	<0.0001 (T1, T2>T0)
Nuclear Intensity Sum (AU)	2,440,722 ± 641,465	4,707,289 ± 73,993	4,718,524 ± 137,875	<0.0001 (T1, T2>T0)

* *Cell and nuclear roundness are proportional to the square root of the area divided by the circumference: it is normalized to give 1 for a perfect circle and decreases for elongated objects. AU, Arbitrary Units. All data are expressed as mean ± SD of measurements performed at single-cell level (cell number>5,000 for T0 and T1 groups, and >3,000 for T2 group)*.

Differentiation-associated modifications of mitochondrial mass of control mononuclear cells were also accompanied by mitochondrial texture changes (Figure 3C). In the T0–T1 interval, the major variations of SER features concern the spot and hole filters, whose indexes reduce by around 18%, and the edge filter, which increases by 13%. This pattern suggests a relative increase of fragmentation over elongation in the mitochondrial architecture. Instead in the T1–T2 interval, SER feature variations suggestive of mitochondrial fragmentation, i.e., decrease of spot, hole, and saddle, are quantitatively similar to the elongation-associated features (ridge and edge), thus indicating a balance in the mixed morphology of mitochondrial network. Mitochondrial texture modifications accompanying STIM1 L96V mononuclear cell differentiation resemble those of control cells in both T0–T1 and T1–T2 intervals. However, the variations of SER indexes indicative of network fragmentation are less pronounced than those of control cells in the T0–T1 interval, and a parallel increase of the saddle and valley features indicates a mitochondrial elongation (Figure 3D).

#### Cell Morphometry and Mitochondrial Texture Properties of Control and STIM1 L96V Multinuclear Cells (T2)

A comparison of morphological features and mitochondrial architecture of multinuclear control and STIM1 L96V cells is reported in Table 4 and Figure 3B, respectively. Multinuclear STIM1 L96V cells are smaller than corresponding normal cells. Curiously, STIM1 L96V cells are also more spindle-shaped, this feature being present during the entire differentiation process (Tables 2, 3). The nuclear parameters of STIM1 L96V cells are all lower than those of control cells, according to the reduced number of nuclei in multinuclear STIM1 L96V cells. The mitotracker mean intensity of STIM1 multinuclear elements is slightly lower than that of control cells, suggesting a reduction of mitochondrial membrane potential. Interestingly, mitotracker mean intensity of STIM1 L96V multinuclear elements was higher than that of the corresponding mononuclear cells at T2, suggesting a fusion-associated increase of mitochondrial function. Mitochondrial texture features of multinuclear control and STIM1 L96V myotubes are reported in Figure 3B. The major variations concern saddle, valley, and hole features, indicating an elongated architecture, even though the increase of ridge, and edge indexes also denotes an increase of the fragmented phenotype.

**Table 4:** Morphological features of control and STIM1 L96V multinuclear cells (T2).

**Cell morphometry**	**Control multinuclear cells**	**STIM1 L96V multinuclear cells**	***P-*value (unpaired *t-*test)**
Cell area [μm^2^]	3,906 ± 425	3,094 ± 892	<0.0001
Cell roundness^*^	0.3302 ± 0.0559	0.2643 ± 0.0461	<0.0001
Mito intensity Mean (AU)	408 ± 59	345 ± 51	<0.0001
Mito intensity Sum (AU)	19,191,957 ± 2,295,938	13,170,555 ± 5,595,763	<0.0001
Nuclear area [μm^2^]	791 ± 139	669 ± 107	<0.0001
Nuclear roundness^*^	0.7031 ± 0.042	0.6895 ± 0.0365	0.02
Nuclear Intensity Mean (AU)	2,652 ± 279	2,074 ± 235	<0.0001
Nuclear Intensity Sum (AU)	36,364,900 ± 10,586,040	23,063,770 ± 5,622,289	<0.0001

* *Cell and nuclear roundness are proportional to the square root of the area divided by the circumference: it is normalized to give 1 for a perfect circle and decreases for elongated objects. AU, Arbitrary Units. All data are expressed as mean ± SD of measurements performed at single-cell level (n > 300 for both control and STIM1 L96V multinuclear cells)*.

### Gene Expression Analysis

#### Genes Involved in Calcium Homeostasis and TA Formation

**Figure 4:**
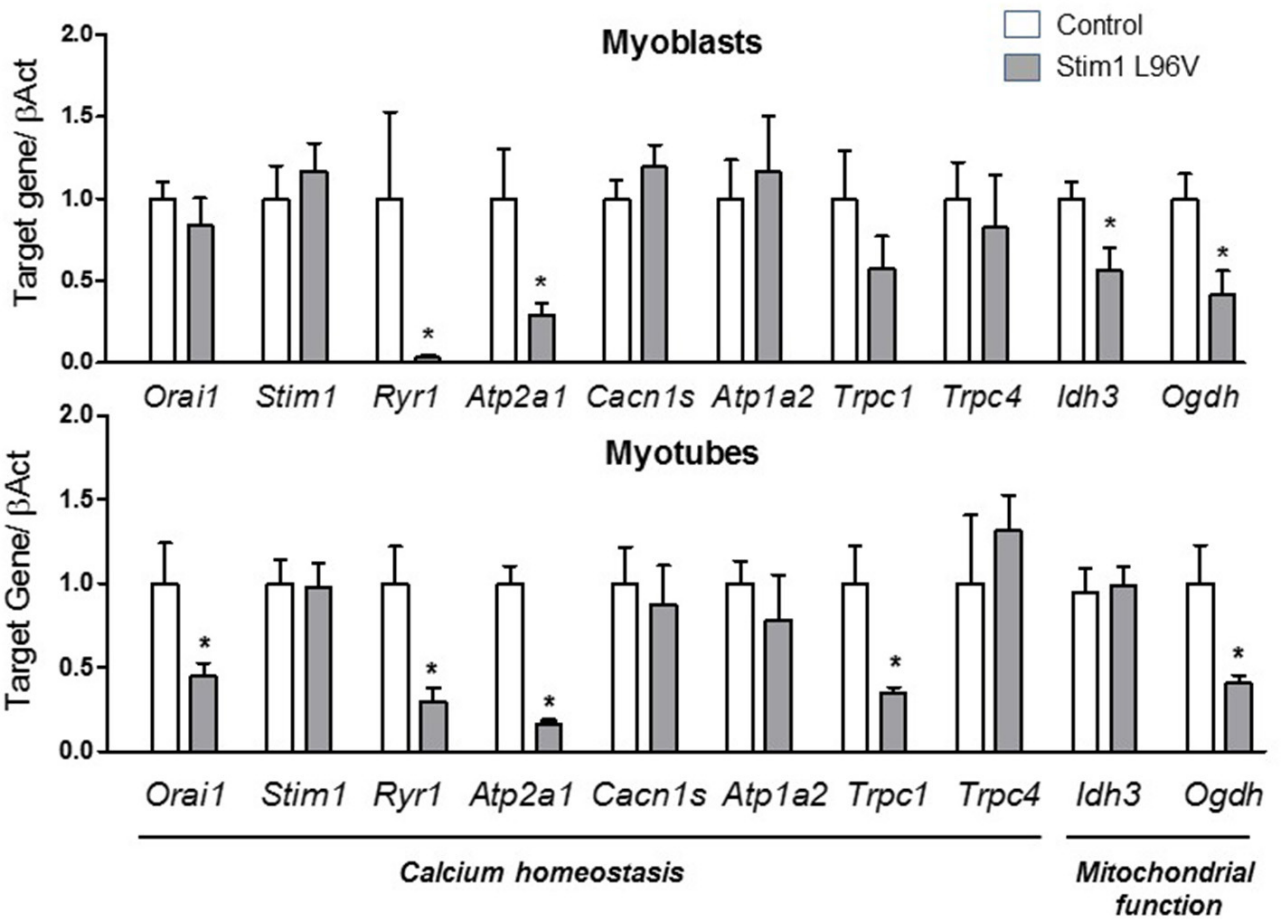
Expression levels of selected genes involved in calcium homeostasis and mitochondrial function. The histograms show the relative content of transcript levels for *Orai1, Stim1, Ryr1, Atp2a1, Cacn1s, Atp1a2, Trpc1, Trpc4* genes involved in calcium homeostasis and *Ogdh*, and *Idh3* involved in mitochondrial function normalized to β*-actin* gene in STIM1 L96V myoblasts (upper Panel) and myotubes (bottom Panel) with respect of control cells. Data are expressed as fold-difference compared with control cells; samples were analyzed in triplicate, and results are expressed as the means ± sem. Statistical significance was determined by unpaired Student's *t-*test, with a value of *P* < 0.05 considered significant, ^*^Significantly different.

Tubular aggregates are displayed in patient derived muscle cells used in our study (Böhm et al., 2014). Different proteins involved in the uptake and Ca^2+^ storage such as STIM1, sarcoplasmic reticulum Ca^2+^-ATPase (SERCA1a) or ryanodine receptor 1 molecule (RyR1) were previously shown to be components of the aggregates (Chevessier et al., 2005; Böhm et al., 2013). In addition, STIM1 could directly interact with other proteins, such as the canonical-type transient receptor potential cationic channels (TRPCs) or the dihydropyridine receptor (DHPR) (Kiselyov and Patterson, 2009; Lee et al., 2013). By qPCR, we found a significant down-regulation of mRNA level of *RyR1* and *Atp2a1* (encoding for SERCA1a) in myoblasts and myotube carrying STIM1 L96V mutation compared to control myoblasts and myotubes, respectively (Figure 4). Any significant change was detected in the expression level of *Stim1, Cacna1s* (encoding for DHPR)*, Atp1a2* (encoding for Na/K ATPase) and *Trpc4*. Importantly, a trend of reduction of *Trpc1* already observed in myoblasts carrying STIM1 L96V mutation, became significant in differentiated myotubes, while *Orai1* expression, resulted unchanged in myoblasts, was significantly reduced in myotubes carrying STIM1 L96V (Figure 4).

#### Genes Involved in Mitochondrial Function

To assess if STIM1 L96V mutation could affect mitochondrial function, we analyzed mRNA expression level of two mitochondrial Ca^2+^-sensitive dehydrogenases fundamental to generate NADH needed by the respiratory chain to generate ATP, such as an isoform of isocitrate dehydrogenases (*IDH3A*), which catalyzes the oxidative decarboxylation of the isocitrate in α-ketoglutarate, and 2-oxoglutarate dehydrogenase (*OGDH*), a component of the α-ketoglutarate dehydrogenase complex that converts α-ketoglutarate to succinate (Denton and McCormack, 1980). A significant reduction of the expression of *IDH3A* and *OGDH* was detected in myoblasts carrying STIM1 L96V mutation with respect to control myoblasts, which is however observed only for *OGDH* in mutated differentiated myotubes (Figure 4).

#### Genes Involved During the Differentiation of Myoblasts to Myotubes

High content imaging analysis revealed a defective myogenesis associated to STIM1 L96V mutant muscle cells with respect to control cells. On this basis, we gained insight into the mechanism underlying the myogenic pathway alteration by analyzing the mRNA levels in STIM1 L96V myoblasts and myotubes of the following genes: *Pax7* (paired box 7), which is a member of the upstream regulators of myogenesis and a marker of satellite cells; *Myf5* (Myogenic factor 5) and *MyoD1* (Myogenic differentiation 1), which are members of the myogenic regulatory factor (MRF) family; *Mef* 2D (Myocyte enhancer factor 2D), which is a member of the myocyte enhancer factor 2 family (MEF2) and *Myog* (Myogenin), both markers of differentiation; finally, *Tnnt3* (Troponin), and *DMD* (Dystrophin), were chosen as markers of late differentiation.

**Figure 5:**
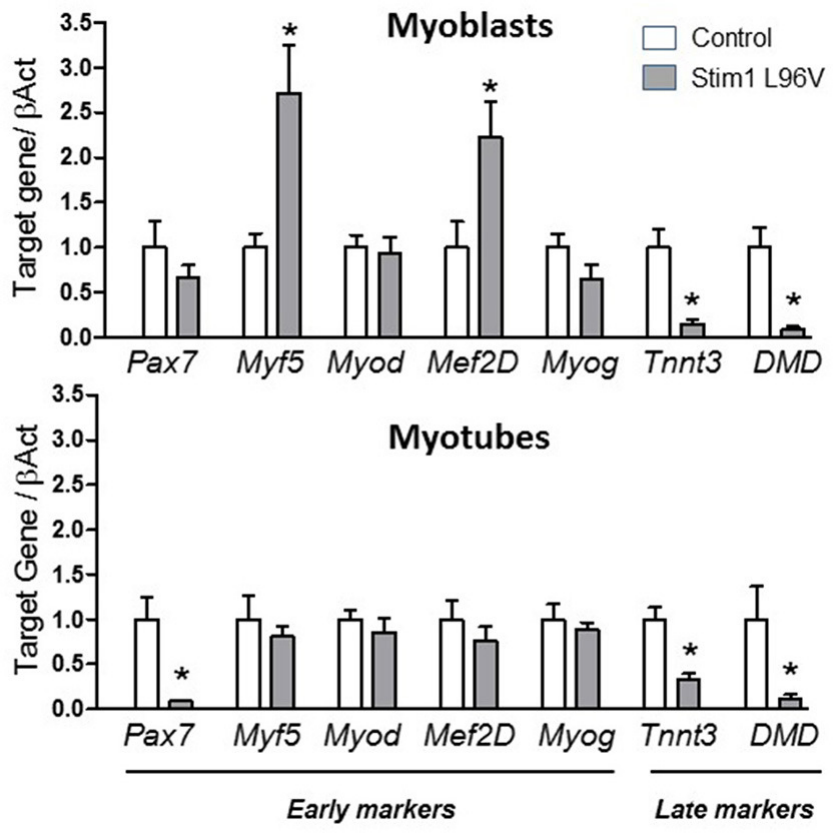
Expression levels of selected genes involved in muscle differentiation. The histograms show the relative content of transcript levels for early markers of differentiation *Pax7, Myf5, Myod, Mef2D* and *Myog* genes, and later markers of differentiation *Tnnt3, DMD* genes normalized to β*-actin* gene in STIM1 L96V myoblasts (upper Panel) and myotubes (bottom Panel) with respect of control cells. Data are expressed as fold-difference compared with control cells; samples were analyzed in triplicate, and results are expressed as the means ± sem. Statistical significance was determined by unpaired Student's *t-*test, with a value of *P* < 0.05 considered significant, ^*^Significantly different.

Interestingly, in STIM1 L96V myoblasts we found a significant reduction in *DMD, Tnnt3* and an increase in *Myf5* and *Mef2D* mRNA levels with respect to control myoblasts. A significant reduction in *Pax7, DMD*, and *Tnnt3* was observed in mutated myotubes with respect to control ones (Figure 5). No significant alteration was observed for *MyoD1* and *Myog* expression levels both in STIM1 L96V myoblasts and myotubes with respect to control cells.

All these findings indicate an altered myogenic pathway associated to STIM1 L96V mutation.

## Discussion

TAM is a rare hereditary myopathy actually without a cure caused by mutations of genes involved in Ca^2+^ homeostasis. In more detail, gain-of-function mutations of STIM1 or ORAI1 genes inappropriately activate the SOCE process and induce an excessive extracellular Ca^2+^ entry despite repleted Ca^2+^ stores (Lacruz and Feske, 2015; Lee and Noguchi, 2016; Böhm and Laporte, 2018; Morin et al., 2020). Ca^2+^ homeostasis alterations affect a variety of cell functions, being Ca^2+^-dependent signaling involved in multiple cellular processes (Carafoli and Krebs, 2016). As far as skeletal muscle cells are concerned, the Ca^2+^-mediated coupling of excitation and contraction has long been well-established, but the recognition of Ca^2+^ relevance in muscle formation, growth and regeneration is also growing (Tu et al., 2016). Most of the STIM1 mutations responsible for TAM are located in the luminal Ca^2+^-sensing EF-hand domain, and affect amino acids thought to be involved in Ca^2+^ coordination or maintaining the protein in a folded and inactive conformation (Böhm and Laporte, 2018). The patients carrying EF-hand mutations are mainly characterized by a muscle phenotype, a proximal muscle weakness being generally reported. However, a precise genotype/phenotype correlation is still undefined, the onset and severity of muscle involvement being not uniform for the different STIM1 EF-hand mutations (Morin et al., 2020). Symptoms vary greatly from patient to patient, with a wide phenotypical spectrum ranging from childhood-onset muscle weakness to adult-onset myalgia. Functional effects of STIM1 mutations at cellular level have been investigated mainly in heterologous expression systems and, only in the case of STIM1 A84G (Böhm et al., 2013) and G81A (Walter et al., 2015) mutations, also in patient- derived myoblasts.

In this study, we have investigated the Ca^2+^ homeostasis alterations of skeletal muscle cells from a TAM patient carrying the STIM1 L96V mutation. This mutation, along with the more recently reported L92V (Morin et al., 2020), is located in the hydrophobic cleft which contributes to keeping the STIM1 inactive conformation, according to molecular modeling simulations (Schober et al., 2019). We confirm in human myoblasts the STIM1 L96V-dependent Ca^2+^ overload already reported in engineered murine myoblasts and detect the Ca^2+^ alteration persistence also in differentiated cells. The resting Ca^2+^ level is set by the balance between influx and efflux mechanisms at rest (Li et al., 2010; Ríos, 2010) and whether STIM1 L96V is the main source for the resting Ca^2+^ dysregulation remain to be defined. Indeed, this is an unsolved aspect also for other described STIM1 mutants (Morin et al., 2020). In this regards, the use of SOCE inhibitors at different times of myogenesis process would be useful to explore the observed differences in resting Ca^2+^ level between patient-derived muscle cells and control cells. Notably, in view of the non-specific effects mediated by SOCE inhibitors actually available (Le Guilcher et al., 2020; Meizoso-Huesca and Launikonis, 2021), focused studies will be required based on the use of several tools to unequivocally solve this issue.

The measured excess of intraluminal and cytosolic Ca^2+^ is accompanied by expression profile modifications of genes involved in Ca^2+^ homeostasis. Our findings of expression profile regarding genes encoding proteins involved in Ca^2+^ homeostasis indicate an adaptive or compensatory response of the cells to the increased SOCE, which was already observable in myoblast and became clear in differentiated myotubes. Indeed, the significant reduction of *Orai1, RyR1*, and *Atp2a1* expression could be considered an attempt of muscle cells to counteract the Ca^2+^ homeostasis dysfunction. The parallel reduction of *Trpc1* could corroborate the involvement of these cationic channels into the STIM1-induced aggregate composition, as reported in other studies (Kiselyov and Patterson, 2009; Lee et al., 2013). Futhermore, RyR1 expression reduction could be also correlate with the expression of the other RyR isoform expressed in skeletal muscle, i.e., RyR3. Indeed, RyR3 isoform has a significant effect on resting Ca^2+^ levels and a precise balance between RyR1 and RyR3 expression physiologically tightly regulate the diversity of cellular responses that muscle cells undergo during their early development (Protasi et al., 2000; Perez et al., 2005). Particularly, in myotubes RyR3 has virtually no role in initiating or maintaining EC coupling. Thus, it may be postulated that the reduction of RyR1 gene expression we observed in Stim1 mutant myotubes is likely related with a consequent RyR1 and RyR3 expression imbalance. This could be supported by the increased level of resting Ca^2+^ as well as by the significantly increased responsiveness to caffeine in STIM1 mutant muscle cells with respect to control cells. Indeed, it has been reported that altered expression of RyR3 resulted in myotubes with significantly higher resting Ca^2+^ level as well as with a different caffeine sensitivity than myotubes expressing RyR1 (Perez et al., 2005).

The STIM1/ORAI1-mediated SOCE is emerging as a critical process in regulating long-term muscle functions and a growing evidence supports its relevance in muscle differentiation, development, and growth (Louis et al., 2008; Darbellay et al., 2009, 2010; Michelucci et al., 2018). To evaluate the cellular response to Ca^2+^ dyshomeostasis, in this study, we compared the morphological features of STIM1 L96V myoblasts and control myoblasts, and investigated the effects of STIM1 L96V mutation on *in vitro* myogenic differentiation process through high content imaging and gene expression analysis. Cells morphology and mitochondrial network are critical for several biological processess that control nuclear programs and are strictley related to Ca^2+^ handeling in skeletal muscle (Favaro et al., 2019). Thus, we particularly focused on these cellular features. First of all, high content imaging was employed for evaluating similarities and differences between control and STIM1 L96V myoblasts at T0, i.e., when differentiation medium was substituted for growth medium. The variability of cellular shape and dimension of both control and STIM1 L96V myoblasts at T0 indicates that cells are in various growth stages and that some of them probably already started the differentiation process. This is most likely related to the use of non-synchronized cell cultures, an ex-ante choice undertaken with the aim of investigating differentiating muscle cells with minimal manipulation. Interestingly, STIM1 L96V myoblasts are larger and more splindle-shaped than control cells. More generally, the morphological features of STIM1 L96V myoblasts at T0 reflect the cellular adaptive response to the STIM1 mutation. In this respect, mitochondria might also be involved in cellular response to Ca^2+^ dyshomeostasis (Bagur and Hajnóczky, 2017). Mitochondria are dynamic organelles characterized by high mobility as well as shape changes, therefore they exhibit a mixed morphology, with small particles and tubular and highly networked structures (Karbowski and Youle, 2003). Organizational changes of mitochondrial morphology are controlled by multiple processes, including biogenesis and fusion and fission events, which adapt mitochondrial shape to the cell physiological needs. Mitochondrial fusion and fission could be related to STIM1-mediated intracellular Ca^2+^ movements and SOCE and alterations of STIM1 expression or activity is associated with mitochondrial abnormalities in skeletal muscle cells (Goonasekera et al., 2014; Choi et al., 2019). SER texture analysis of mitochondrial network indicate that STIM1 L96V myoblasts at T0 are characterized by a more elongated and networked mitochondrial architecture with respect to control myoblasts. The Ca^2+^ overload associated with STIM1 L96V mutation could prematurely activate and/or upregulate the Ca^2+^-dependent pathways, thus inducing an earlier onset of the differentiation process. This possibility is supported by the early appearance of spindle-shaped STIM1 L96V cells as well as by their more marked spindle-shaped morphology. If that was the case, it should be hypothesized that the mitochondrial system develops alongside the SR and myofibrils, as suggested by the elongated mitochondrial architecture observed in STIM1 L96V myoblasts in the early phase of differentiation.

High content imaging analysis of the differentiation process of control myoblasts shows a progressive increase of cell size and splindle-cell morphology. The parallel increase of mitochondrial mass and concentration also suggests an enhancement of mitochondrial activity. Mitotraker deep red accumulation is indeed dependent upon mitochondrial membrane potential (Poot et al., 1996), and an increase of mitotracker signal intensity associated with myogenic differentiation has already been reported (Miyake et al., 2011). Differentiation-associated modifications of mitochondrial mass of control myoblasts are also accompanied by mitochondrial network modifications, the SER texture analysis likely indicating an initial prevalence of mitochondrial fragmentation followed by a balance condition of fragmented/elongated architecture. Indeed, the mitochondrial network SER modifications of differentiating control myoblasts are in agreement with results reported by Sin et al. (2016), who showed a mitochondrial dynamic remodeling in differentiating C2C12 myoblasts, characterized by a network fragmentation in the early phases of the process. Interestingly, the mitochondrial architecture modifications here reported could represent the morphological counterpart of the mitochondrial function modifications associated to human muscle cell differentiation (Hoffmann et al., 2018). In view of the well-established dysfunction of SR structure in TAM (Chevessier et al., 2005; Böhm et al., 2013, 2017; Morin et al., 2020), we cannot not rule out that mitochondrial alterations we observed herein are a secondary phenomenon to the altered SR functionality.

The STIM1 L96V mutation affects the myoblast differentiation process, the most striking differences with respect to control myoblasts being the longer persistence of mononuclear cells along with the formation of multinuclear elements with reduced size, mitochondrial mass and concentration, and nuclei number. Overall, these findings indicate that STIM1 L96V mutation and the associated Ca^2+^ overload induce a complex cell response affecting the myoblast differentiation program. In particular, the alterations of mitochondrial mass and texture along with the reduced expression of IDH3A and OGDH, two key genes of mitochondrial metabolism, strongly suggest a mitochondrial dysfunction associated with STIM1 L96V mutation. Interestingly, a recent reported mouse model bearing a gain-of-function mutation in STIM1 displays histological and muscle alteration associated with mitochondria dysfunction evidenced by the presence of enlarged mitochondria with abnormal morphology (Cordero-Sanchez et al., 2019).

A major phenotypic effect of STIM1 L96V mutation upon myoblast differentiation is represented by the reduced formation of multinuclear elements, this suggesting a STIM1 L96V-dependent delay in the fusion process. A critical event in muscle formation during both embryonic development and regeneration upon injury, is the fusion of myoblasts into multinucleated myotubes. Molecular and cellular mechanisms of myoblast fusion are less known than those of preceding events, even though significant advances have been recently achieved. Cell fusion is a complex event that requires the coordination of various processes culminating in the activation of dedicated proteins, named fusogens, responsible for mediating membrane fusion (Hernández and Podbilewicz, 2017; Sampath et al., 2018). It is generally acknowledged that the fusion of myoblasts into multinucleated myotubes is regulated by calcium-dependent signaling. In particular, the increase of intracellular Ca^2+^ determines the calcineurin-mediated activation of NFAT transcription factor and myoblast fusion (Hindi et al., 2013). However, coordination and crosstalk of many signaling pathways are involved in myoblast fusion, and a complete picture of underlying cellular events is still lacking. In this context, it cannot be excluded that also a reduction of intracellular Ca^2+^ could be at some point required for cell fusion, as in the case of choriocarcinoma BeWo cells (Vatish et al., 2012), and that this event could be compromised by the STIM1 L96V-dependent Ca^2+^ overload.

By assessing the gene expression of some key myogenic factors leading to cell differentiation and fusion into multinucleated myotubes, we confirm the finding of our morphological imaging analysis highlighting an alteration of the myogenic pathway associated to TAM mutant. Particularly the gene expression increase of early differentiation markers such as *Myf5* and *Mef2D* together with the reduction of late differentiation markers such as *DMD and Tnnt3*, encoding for dystrophin and troponin, in mutant myoblasts and myotubes strongly supported an altered myogenesis associated to TAM mutant, mainly regarding the late differentiation phase. In comparison with respect to control cells, STIM1 Leu96Val myoblasts differentiation into myotubes is early started but is not concluded. Future focused studies aimed to detect the protein expression levels of the genes of interest will certainly contribute to gain further insight into the role of these differentiation biomarkers.

### Cellular Models for Personalized Therapeutics

To date, there is no specific treatment recommended for TAM patients. At least in principle, TAM could potentially benefit from treatment with SOCE/CRAC channel inhibitors, a group of putative immunomodulatory agents proposed for some chronic immune-related disorders (Riva et al., 2018; Stauderman, 2018). As far as preclinical investigations are concerned, there are two reported murine models bearing STIM1 gain-of function mutations and exhibiting a muscular phenotype (Gamage et al., 2018; Silva-Rojas et al., 2019), and only one is characterized by the luminal EF-hand mutation I115F (Cordero-Sanchez et al., 2019). In this respect, in our study, besides highilighting new etiopathological mechanisms underlying TAM disease, we validated a preclinical cellular model for TAM. We believe that preclinical investigations could benefit also from a preliminary phenotypic *in vitro* screening based upon the findings here reported, i.e., upon the ability of candidate SOCE/CRAC inhibitors to counteract the differentiation alterations associated with Ca^2+^ overload. As it is usually desired for neuromusculuar disorders (Silva-Rojas et al., 2020; van Putten et al., 2020), a such experimental approach could finally allow a reliable translation in the clinical management of TAM patients.

## Data Availability Statement

The raw data supporting the conclusions of this article will be made available by the authors, without undue reservation.

## Ethics Statement

The studies involving human participants were reviewed and approved by human muscle samples were provided by the Telethon biobank at Besta Neurological Institute in Milan. Research was conducted according to protocols approved by the Institutional Review Board of the Besta Neurological Institute and University of Bari, and in compliance with the Helsinki Declaration and local legislation. The patients/participants provided their written informed consent to participate in this study.

## Author Contributions

EC and AL conceived and coordinated the study. LM, MM, and SG provided the muscle biopsy. PI and SG performed cell culture. EC and AL performed calcium cytofluorimetry experiments. AP and MC performed high content imaging experiments. EC and GC performed quantitative PCR experiments. PI, OC, and AD contributed ideas and critically interpreted results. EC, AP, MC, and AL wrote the paper. All authors approved the final version of the manuscript.

## Conflict of Interest

The authors declare that the research was conducted in the absence of any commercial or financial relationships that could be construed as a potential conflict of interest.
