# Supplementary material for: Gain-of-Function STIM1 L96V Mutation Causes Myogenesis Alteration in Muscle Cells From a Patient Affected by Tubular Aggregate Myopathy
Source: Front Cell Dev Biol. 2021 Feb 26;9:635063. doi: 10.3389/fcell.2021.635063 (PMC7952532; doi:10.3389/fcell.2021.635063)
Supplement: Supplementary file 1 [file Data_Sheet_1.PDF]

Supplementary material

Genes involved during the differentiation of myoblasts to myotubes: analysis in an additional control cell line (HMb_2)

To ensure that the cell deriving from patient not TAM affected (Control), used here *versus* and compared STIM1 mutant cells, could likely represent a reference control patient cell line resembling “control features” for muscle miogenesis, we compare its gene expression profile regarding myogenesis with the related gene expression profile of another human control cell line. Particularly, we used human derived myoblasts from a healthy donor, age matched with TAM patient and not TAM affected patient (HMb_2). Importantly, no significant difference was observed between the two control cell lines both in myoblasts and myotubes (Figure 1S).

Furthermore, human derived fibroblast, used as negative control for myogenesis related genes, showed no expression of the reported genes, with the exception of Mef2, only expressed by about 20% with respect to control myoblasts (data not shown).

High Content Imaging: mitochondrial texture analysis

The mitochondrial texture properties were analyzed using the SER features building block of the Harmony software. Briefly, the image texture features usually described as smooth, rough, granular, homogeneous/inhomogeneous, linear *etc*. have been quantified calculating the numerical properties which quantitatively describe the texture. The SER (Spot, Edge, Ridge) features method includes a set of eight properties (spot, hole, edge, ridge, valley, saddle, bright and dark) sensitive to distinct intensity patterns according to the property geometry designation.

Briefly, the original images were passed through a set of six filters, each of which is based on gradient and curvature estimates for each pixel and can be used to describe the intensity landscapes in the pixel neighborhood. The set of six intensity patterns that were searched for in the mitotracker image region is shown in Figure S2 (a). By way of example, the original images of two myoblasts at T0, a normal and a STIM1 L96V myoblast, are shown in Figure S2 (b) and (c), respectively, and the corresponding SER-filtered images and SER values are shown in the panels below. The spatial distribution of intensity of the cytoplasmic region of control and STIM1 L96V myoblasts, *i.e*. their mytocondrial architecture, was quantitatively described by the frequency of a specific feature or combination of features, and the results are shown in Figure S2 (d). The results show that all SER texture indexes of STIM1 L96V myoblasts were significantly different from those of control myoblasts, except for spot and hole feature, indicating a difference of mitochondrial network organization. The major differences concern in the order saddle, valley, edge and ridge filters, indicating that STIM1 L96V myoblasts at T0 are characterized by a more elongated and networked mitochondrial architecture with respect to control myoblasts.

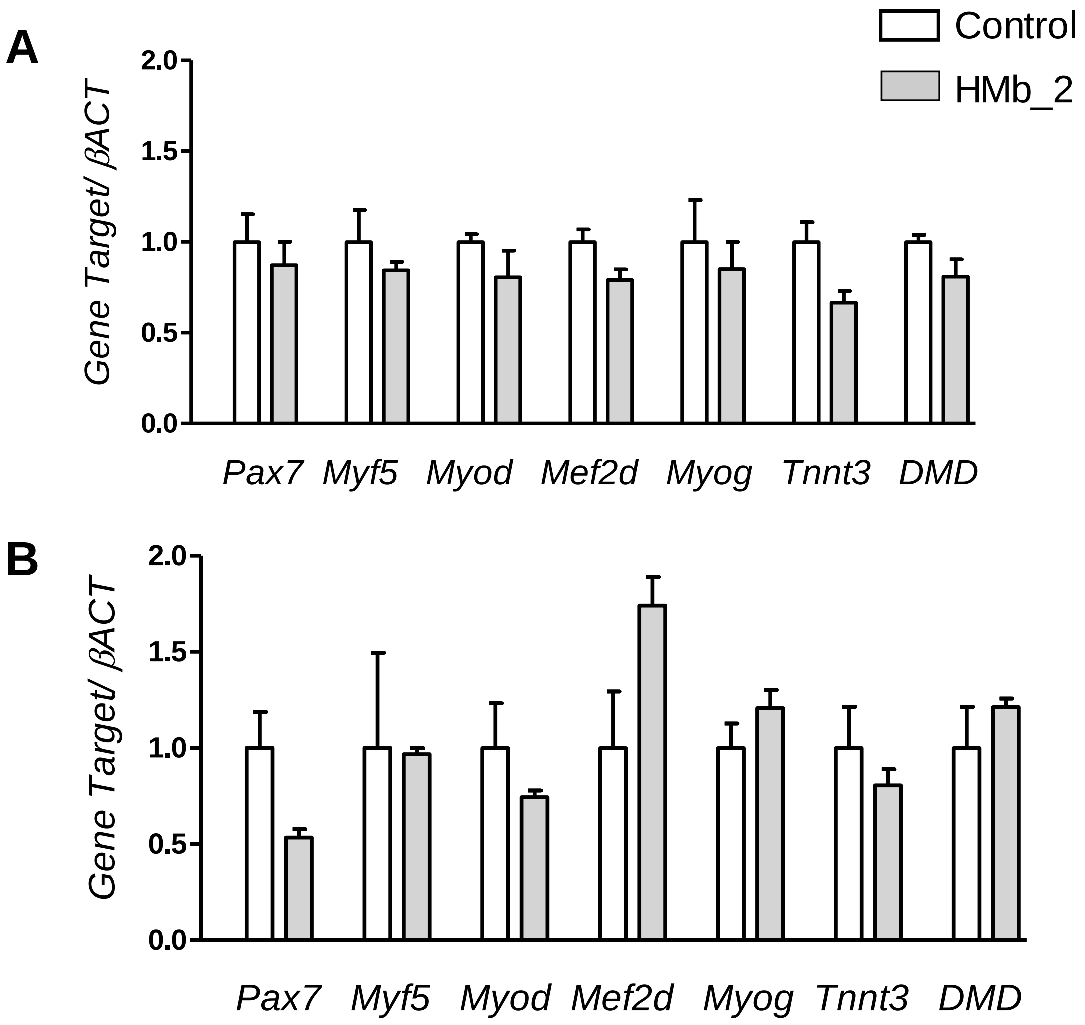

Figure S1. Myogenesis genes expression in myoblasts and myotubes from two healthy patients. The histograms show the relative content of transcript levels for *Pax7*, *Myf5*, *Myod*, *Mef2D*, *Myog*, *Tnnt3*, *DMD* genes normalized to β-actin gene in myoblasts (**A**) and myotubes (**B**) derived from non TAM affected control patient (use in the study as a reference Control cells) and a control muscle cell line (HMb_2). Data are expressed as fold-difference compared with non TAM control patient derived muscle cells; samples were analyzed in triplicate, and results are expressed as the means ± sem. Statistical significance was determined by unpaired Student's t test, with a value of $P < 0.05$ considered significant.

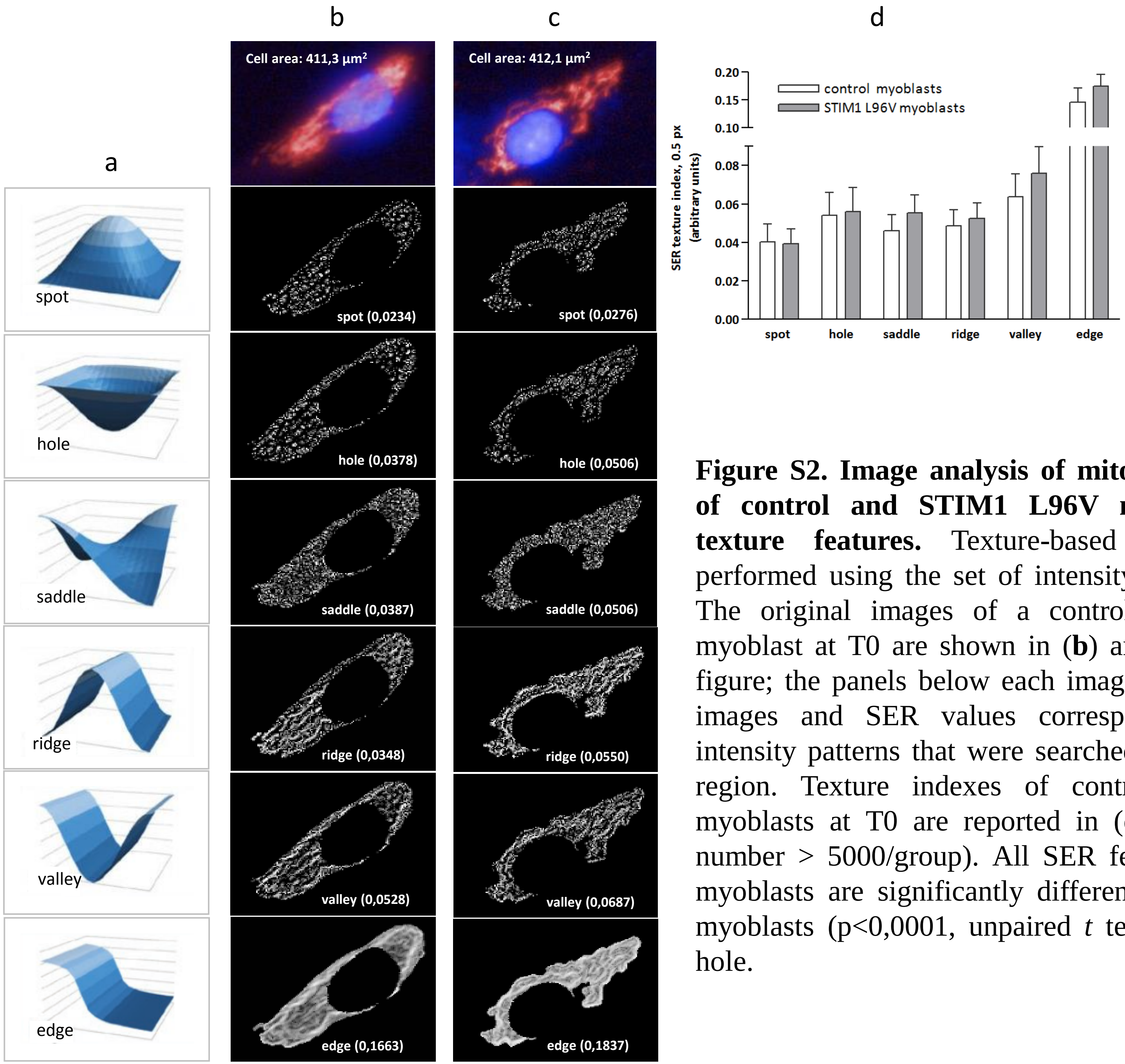

Figure S2. Image analysis of mitochondrial architecture of control and STIM1 L96V myoblasts, using SER texture features. Texture-based image analysis was performed using the set of intensity patterns shown in (**a**). The original images of a control and a STIM1 L96V myoblast at T0 are shown in (**b**) and (**c**), at the top of the figure; the panels below each image show the SER-filtered images and SER values corresponding to the distinct intensity patterns that were searched for in the cytoplasmic region. Texture indexes of control and STIM1 L96V myoblasts at T0 are reported in (**d**) as mean ± SD (cell number > 5000/group). All SER features of STIM1 L96V myoblasts are significantly different from those of control myoblasts (p<0,0001, unpaired *t* test), except for spot and hole.
